# VENTILatOry strategies in patients with severe traumatic brain injury: the VENTILO Survey of the European Society of Intensive Care Medicine (ESICM)

**DOI:** 10.1186/s13054-020-02875-w

**Published:** 2020-04-17

**Authors:** Edoardo Picetti, Paolo Pelosi, Fabio Silvio Taccone, Giuseppe Citerio, Jordi Mancebo, Chiara Robba, on the behalf of the ESICM NIC/ARF sections

**Affiliations:** 1 Department of Anesthesia and Intensive Care, Parma University Hospital, Via Gramsci 14, 43100 Parma, Italy; 2 Department of Anesthesia and Intensive Care, IRCCS for Oncology and Neurosciences, Genoa, Italy; 3 Department of Surgical Sciences and Integrated Diagnostics, University of Genoa, Genoa, Italy; 4 Department of Intensive Care, Erasme Hospital, Université Libre de Bruxelles (ULB), Brussels, Belgium; 5 School of Medicine and Surgery, University of Milan – Bicocca, Monza, Italy; 6 Department of Intensive Care, Sant Pau Hospital, Barcelona, Spain

**Keywords:** Traumatic brain injury, Mechanical ventilation, Respiratory failure

## Abstract

**Background:**

Severe traumatic brain injury (TBI) patients often develop acute respiratory failure. Optimal ventilator strategies in this setting are not well established. We performed an international survey to investigate the practice in the ventilatory management of TBI patients with and without respiratory failure.

**Methods:**

An electronic questionnaire, including 38 items and 3 different clinical scenarios [arterial partial pressure of oxygen (PaO_2_)/inspired fraction of oxygen (FiO_2_) > 300 (scenario 1), 150–300 (scenario 2), < 150 (scenario 3)], was available on the European Society of Intensive Care Medicine (ESICM) website between November 2018 and March 2019. The survey was endorsed by ESICM.

**Results:**

There were 687 respondents [472 (69%) from Europe], mainly intensivists [328 (48%)] and anesthesiologists [206 (30%)]. A standard protocol for mechanical ventilation in TBI patients was utilized by 277 (40%) respondents and a specific weaning protocol by 198 (30%). The most common tidal volume (TV) applied was 6–8 ml/kg of predicted body weight (PBW) in scenarios 1–2 (72% PaO_2_/FIO_2_ > 300 and 61% PaO_2_/FiO_2_ 150–300) and 4–6 ml/kg/PBW in scenario 3 (53% PaO_2_/FiO_2_ < 150). The most common level of highest positive end-expiratory pressure (PEEP) used was 15 cmH_2_O in patients with a PaO_2_/FiO_2_ ≤ 300 without intracranial hypertension (41% if PaO_2_/FiO_2_ 150–300 and 50% if PaO_2_/FiO_2_ < 150) and 10 cmH_2_O in patients with intracranial hypertension (32% if PaO_2_/FiO_2_ 150–300 and 33% if PaO_2_/FiO_2_ < 150). Regardless of the presence of intracranial hypertension, the most common carbon dioxide target remained 36–40 mmHg whereas the most common PaO_2_ target was 81–100 mmHg in all the 3 scenarios. The most frequent rescue strategies utilized in case of refractory respiratory failure despite conventional ventilator settings were neuromuscular blocking agents [406 (88%)], recruitment manoeuvres [319 (69%)] and prone position [292 (63%)].

**Conclusions:**

Ventilatory management, targets and practice of adult severe TBI patients with and without respiratory failure are widely different among centres. These findings may be helpful to define future investigations in this topic.

## Background

Traumatic brain injury (TBI) is a worldwide health problem with elevated rate of mortality and disability [1]. Brain-injured patients with altered consciousness frequently require intubation and invasive mechanical ventilation to protect the airways from aspiration and to prevent harmful secondary insults, such as hypoxemia [generally defined as an arterial partial pressure of oxygen (PaO_2_) < 60 mmHg] and hypercapnia [generally defined as an arterial partial pressure of carbon dioxide (PaCO_2_) > 45 mmHg] [2]. TBI patients may develop severe respiratory failure and acute respiratory distress syndrome (ARDS) during intensive care unit (ICU) stay [3, 4]. Lung-protective ventilation strategies, with low tidal volumes (TVs) and moderate-to-high positive end-expiratory pressure (PEEP), are associated with improved outcomes in ARDS and non-ARDS patients [5, 6] and are characterized by “low ranges” oxygenation targets and permissive hypercapnia [7, 8]. This approach, considering its potential adverse cerebrovascular effects, is difficult to apply in TBI patients, regardless of the presence of intracranial hypertension [2, 9, 10]. Traditionally, in TBI patients, low PEEP and high TVs are generally applied for tight CO_2_ control [5]; however, recent evidence suggests that, even in TBI patients, the use of high TVs is associated with the development of acute lung injury [9, 11]. As such, brain-injured patients have been excluded from the major trials exploring the effect of lung-protective ventilation strategies in ARDS [12, 13], and consequently optimal ventilatory strategies have not been established yet in this setting [14, 15]. We therefore performed an international survey with the aim to investigate the practice in the respiratory management of TBI patients, with and without respiratory failure.

## Methods

This international survey was endorsed by the European Society of Intensive Care Medicine (ESICM) and promoted by the Neuro-intensive Care (NIC) and the Acute Respiratory Failure (ARF) sections. An electronic questionnaire, including 38 items and 3 different clinical scenarios [PaO_2_ / inspired fraction of oxygen (FiO_2_) (PaO_2_/FiO_2_) > 300, 150–300, < 150], was available on the ESICM website between November 2018 and March 2019 (Appendix 1 in the Additional file 1). The survey was developed by two investigators (E.P. and C.R.) following a non-systematic review of the literature on respiratory management in TBI patients. The questionnaire was created considering some issues around this topic, such as low levels of evidence, lack of good quality studies and controversial results from observational trials. The survey was designed to identify (a) characteristics of the participants demographics, type of hospital/specialty and available neuromonitoring tools (questions 1 to 10), (b) protocols for mechanical ventilation and weaning (questions 11, 12 and 14) and (c) respiratory management strategies (questions 13 and 15 to 38). The target audience was ESICM members who had agreed to participate in ESICM surveys at the time of their membership registration and who treat patients with TBI in their clinical practice. The investigators invited the target participants to involve more respondents locally. Participants did not receive compensation for their participation in the survey, which was distributed via the ESICM office, thus protecting data confidentiality and anonymity. The survey was registered within the ESICM Survey portfolio and no ethical approval was required. The questionnaire was not specifically tested in a pilot cohort of potential respondents but underwent a peer-review process within the ESICM Research Committee.

### Data analyses and statistical methods

Data from the questionnaire were exported as a comma-separated value report from the Surveymonkey® software package and subsequently stored as an Excel file (Microsoft Corp, Redmond, WA). Descriptive statistics were computed for all study variables. The results are presented as numbers and percentage. Two main subdivisions were considered in the population: one based on geographic area (Europe vs. Others), one based on ICU characteristics [specialized neuro-ICU (NICU) vs. non-specialized NICU]. Results for the population as a whole and for the sub-group subdivision are reported. Differences between groups (e.g. specialized NICU vs. non-specialized NICU, Europe vs. Others) were assessed using a chi-squared test for binary variables in 2 × 2 or r × c contingency tables. Cells with fewer than 5 cases were grouped with other cells taking care that the new group was clinically appropriate. For the questions where more than one test was necessary (for example questions concerning rescue strategies), a Bonferroni correction for multiple comparisons was adopted. Stata software release 13.0 was used for the statistical analysis (StataCorp, 2013, Stata Statistical Software, Release 13; StataCorp LP, College Station, TX). *P* < 0.05 was considered as statistically significant.

## Results

**Fig. 1:**
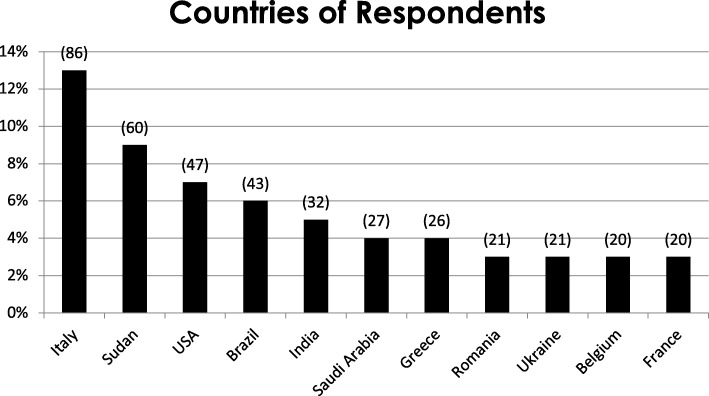
Number of respondents per country. Only countries with a number of responses > 20 have been included. Abbreviations: USA = United States of America. The number of respondents is shown in brackets

The total number of respondents was 687 from 676 centres around the world [the number of respondents in relation to completed items is shown Table S1 in the Additional file 1). Most of the respondents (*n* = 472 [69%]) were from Europe. Italy was the country with the highest number of respondents (*n* = 86), followed by Sudan (*n* = 60), the USA (*n* = 47) and Brazil (*n* = 43) (Fig. 1). The majority of respondents were intensivists (*n* = 328 [48%]) and anesthesiologists (*n* = 206 [30%]) working in mixed general and NICUs (*n* = 278 [41%]). Baseline characteristics of the survey participants are shown in Table 1.

**Table 1:** Baseline characteristics of the overall population

	Respondents (*n* = 687)
**Gender**	
Male	452 (66%)
Female	235 (34%)
**Age (years)**	
< 35	115 (17%)
35–45	282 (41%)
45–65	249 (36%)
> 65	41 (6%)
**Specialty**	
Intensive care medicine	328 (48%)
Anesthesiology	206 (30%)
Neurocritical care	61 (9%)
Internal medicine	35 (5%)
Others	57(8%)
**Post-specialization experience in critical care (years)**	
1–5	180 (26%)
6–10	149 (22%)
> 10	358 (52%)
**Type of ICU**	
Mixed general and neuroICU	278 (41%)
General ICU	271 (39%)
Specialized neuroICU	130 (19%)
Others	8 (1%)
**ICU beds**	
< 5	111 (16%)
6–10	196 (29%)
11–15	133 (19%)
> 15	247 (36%)
**Affiliation**	
University	501 (73%)
Non-university	186 (27%)
**Available bedside neuromonitoring**	
ICP	546 (80%)
PbtO2	149 (22%)
NIRS	215 (31%)
TCD	442 (64%)
Cerebral microdialysis	50 (7%)
Intermittent EEG	522 (76%)
Continuous EEG	262 (38%)
Pupillometer	85 (12%)
SjVO2	209 (30%)

Abbreviations: *ICU* intensive care unit, *ICP* intracranial pressure, *PbtO2* brain tissue oxygen tension, *NIRS* near infrared spectroscopy, *TCD* transcranial Doppler, *EEG* electroencephalography, *SjVO2* jugular venous oxygen saturation

A standard protocol for mechanical ventilation in TBI patients was utilized by 277 (40%) respondents and a specific weaning protocol by 198 (30%). Automated ventilation modes were utilized by 331 (50%) participants. A driving pressure < 15 cmH_2_O and a plateau pressure < 30 cmH_2_O were utilized by most respondents [respectively 436 (68%) and 616 (95%)].

Ventilator settings and respiratory targets utilized in the three clinical scenarios with different respiratory failure severity are reported in Table 2. The most common TV applied was 6–8 ml/kg/predicted body weight (PBW) in case of PaO_2_/FiO_2_ ≥ 150 (72% for PaO_2_/FiO_2_ > 300 and 61% for PaO_2_/FiO_2_ 150–300) and 4–6 ml/kg/PBW in case of PaO_2_/FiO_2_ < 150 (53%). In patients without intracranial hypertension, the most utilized highest PEEP and PaCO_2_ targets in all 3 clinical scenarios were 15 cmH_2_O (30% for PaO_2_/FiO_2_ > 300, 41% for PaO_2_/FiO_2_ 150–300 and 50% for PaO_2_/FiO_2_ < 150) and 36–40 mmHg (51% for PaO_2_/FiO_2_ > 300, 47% for PaO_2_/FiO_2_ 150–300 and 37% for PaO_2_/FiO_2_ < 150) respectively. In patients with intracranial hypertension, the most utilized highest PEEP was 5 cmH_2_O in scenario 1 (27%) and 10 cmH_2_O in scenarios 2–3 (32% for PaO_2_/FiO_2_ 150–300 and 33% for PaO_2_/FiO_2_ < 150) whereas the PaCO_2_ targets were 36–40 mmHg in all 3 scenarios (43% for PaO_2_/FiO_2_ > 300, 49% for PaO_2_/FiO_2_ 150–300 and 47% for PaO_2_/FiO_2_ < 150). The most common PaO_2_ target was 81–100 mmHg in all the 3 groups (57% for PaO_2_/FiO_2_ > 300, 53% for PaO_2_/FiO_2_ 150–300 and 45% for PaO_2_/FiO_2_ < 150).

**Table 2:** Ventilator settings and respiratory targets utilized in the three clinical scenarios

**PaO**_**2**_**/FiO**_**2**_	**TV (ml/kg PBW)**	**Highest PEEP** **no-IH** **(cmH**_**2**_**O)**	**Highest PEEP** **IH** **(cmH**_**2**_**O)**	**PaCO**_**2**_ **no-IH** **(mmHg)**	**PaCO**_**2**_ **IH** **(mmHg)**	**PaO**_**2**_**(mmHg)**	**SpO**_**2**_**(%)**
**1) > 300**	**6–8** [433(72%)] 4–6 [136(23%)] 8–10 [21(3%)] other [11(2%)]	**15** [182(30%)] 10 [158(26%)] 8 [94(15%)] 5 [69(13%)] other [98(16%)]	**5** [163(27%)] 10 [155(26%)] 8 [135(23%)] 15 [52(8%)] other [96(16%)]	**36–40** [308(51%)] 41–45 [105(18%)] any PaCO_2_* [93(16%)] 30–35 [54(9%)] 46–55 [16(2%)] other [25(4%)]	**36–40** [260(43%)] 30–35 [249(41%)] 41–45 [42(8%)] 46–55 [8(1%)] any PaCO_2_* [7(1%)] other [35(6%)]	**81–100** [345(57%)] 55–80 [101(17%)] 101–120 [93(15%)] nmt** [44(7%)] > 120 [18(4%)]	**> 95** [311(52%)] 92–94 [257(43%)] 88–91 [33(5%)]
**2) 150–300**	**6–8** [331(61%)] 4–6 [192(36)] 8–10 [3(1%)] other [10(2%)]	**15** [218(41%)] 10 [136(25%)] 8 [71(13%)] 5 [23(5%)] other [88(16%)]	**10** [171(32%)] 8 [131(24%)] 5 [103(19%)] 15 [54(11%)] other [77(14%)]	**36–40** [250(47%)] 41–45 [110(21%)] any PaCO_2_* [91(19%)] 46–55 [35(6%)] 30–35 [34(6%)] other [20(4%)]	**36–40** [262(49%)] 30–35 [176[33%)] 41–45 [54(9%)] any PaCO_2_* [10(2%)] 46–55 [4(1%)] other [30(6%)]	**81–100** [283(53%)] 55–80 [115(21%)] 101–120 [84(16%)] nmt** [35(6%)] > 120 [19(4%)]	**92–94** [258(48%)] > 95 [228(43%)] 88–91 [50(9%)]
**3) < 150**	**4–6** [252(53%)] 6–8 [203(42%)] 8–10 [9(2%)] other [16(3%)]	**15** [239(50%)] 10 [95(20%)] 8 [37(7%)] 5 [14(3%)] other [96(20%)]	**10** [158(33%)] 8 [94(20%)] 15 [91(19%)] 5 [55(11%)] other [83(17%)]	**36–40** [175(37%)] 41–45 [118(25%)] any PaCO_2_* [87(16%)] 46–55 [48(10%)] 30–35 [29(7%)] other [19(5%)]	**36–40** [224(47%)] 30–35 [129[26%)] 41–45 [75(16%)] any PaCO_2_* [15(3%)] 46–55 [9(2%)] other [29(6%)]	**81–100** [218(45%)] 55–80 [151(32%)] 101–120 [61(13%)] nmt** [36(7%)] > 120 [14(3%)]	**92–94** [227(47%)] > 95 [148(31%)] 88–91 [106(22%)]

*If arterial pH is in range

**Adjusted to

Abbreviations: *TV* tidal volume, *PEEP* positive end-expiratory pressure, *PaCO*_*2*_ partial pressure of arterial carbon dioxide, *PaO*_*2*_ partial pressure of arterial oxygen, *SpO*_*2*_ arterial blood oxygen saturation, *IH* intracranial hypertension, *PBW* predicted body weight, *nmt* neuromonitoring

**Fig. 2:**
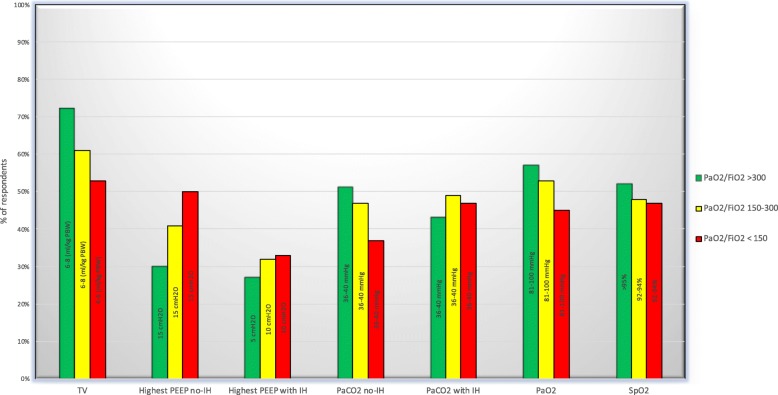
The commonest ventilator settings and respiratory targets in the three clinical scenarios. Abbreviations: TV = tidal volume, PBW = predicted body weight, PEEP = positive end-expiratory pressure, IH = intracranial hypertension, PaCO_2_ = arterial partial pressure of carbon dioxide, PaO_2_ = arterial partial pressure of oxygen, SpO_2_ = arterial blood oxygen saturation, FiO_2_ = inspired oxygen fraction

The most common ventilator settings and respiratory targets in the 3 clinical scenarios are presented in Fig. 2. The most frequent rescue strategies utilized in case of refractory respiratory failure, despite the application of a lung-protective ventilation strategy, were neuromuscular blocking agents (NMBAs) [406 (88%)], followed by recruitment manoeuvres [319 (69%)] and prone position [292 (63%)] (Table 3).

**Table 3:** Rescue strategies utilized in case of refractory respiratory failure

Type of rescue strategy	Respondents (*n* = 464)
- NMBAs	406 (88%)
- Recruitment manoeuvres	319 (69%)
- Prone position	292 (63%)
- Bronchoscopy	239 (52%)
- V-V ECMO	220 (47%)
- NO	115 (25%)
- Extracorporeal CO_2_ removal (DECAP®)	39 (8%)
- Prostacycline	38 (8%)

Abbreviations: *NMBAs* neuromuscular blocking agents, *V-V ECMO* veno-venous extracorporeal membrane oxygenation, *NO* nitric oxide, *CO*_*2*_ carbon dioxide

Data regarding comparisons between European versus non-European respondents and non-specialized versus specialized NICUs are reported in Table S2 in the Additional file 1. European respondents, when compared to non-European, had (1) less frequently standardized protocols for mechanical ventilation and weaning, (2) higher PaCO_2_ targets, (3) a higher utilization of neuromonitoring to set PaO_2_ targets and (4) more frequent use of NMBAs as rescue strategy for refractory respiratory failure.

Respondents working in specialized NICUs, compared to those from non-specialized NICUs, had (1) a less frequent use of plateau pressure target < 30 cmH_2_O and (2) higher PaO_2_ targets.

Data regarding available bedside neuromonitoring between non-specialized versus specialized NICUs are reported in Table S3 in the Additional file 1. Except for near-infrared spectroscopy (NIRS), specialized NICUs, compared to non-specialized NICUs, had more available bedside neuromonitoring tools.

## Discussion

This international survey provides important information regarding the respiratory management of TBI patients admitted to ICU. The main results of our survey can be summarized as follows: (1) few respondents utilized specific protocols for mechanical ventilation and weaning in TBI, (2) low TVs with a PaCO_2_ target of 36–40 mmHg are frequently utilized, (3) lower levels of PEEP are employed in case with intracranial hypertension, (4) NMBAs are the most common rescue strategy in cases of refractory respiratory failure and finally (5) a great variability in practices exists.

To our knowledge, this is the largest survey published so far regarding ventilator strategies in head injured patients. Respondents are coming from different countries around the world, and therefore, our results are representative of the worldwide current clinical practice in this field.

### Ventilator settings and respiratory targets in TBI

Lung-protective ventilation strategies have shown to have a beneficial impact on outcome in patients with and without ARDS [15]. In particular, low TVs are a fundamental component of lung-protective ventilation and their utilization is associated with a reduced mortality in ARDS patients [5, 6]. Although low TVs can potentially cause hypercapnia and consequent intracranial cerebral vessel vasodilation, the use of high TVs in TBI patients (> 9 ml/kg/PBW), with normal lung at ICU admission, is associated with the development of ARDS [11]. Prospective studies regarding low TVs are lacking in TBI and the optimal TV value still need to be established in this setting. Surprisingly, the majority of our respondents declared that they utilize low TVs, even in specialized NICUs, thus suggesting that the concept of lung-protective ventilation is gaining interest even in this group of patients.

The application of PEEP, as well as low TVs, is an important component of lung-protective ventilation [7]. PEEP reduces atelectasis and improves PaO_2_ and lung compliance [7]. Traditionally, low PEEP levels (≤ 5 mmHg) have been utilized in acute brain injury patients admitted to ICU [5], because of the potential risks on cerebral circulation and intracranial pressure (ICP); in particular, the effect of PEEP on ICP seems to be related to both hemodynamic factors and respiratory system compliance [9, 10]. Elevated PEEP levels may reduce systemic venous return, mean arterial pressure and consequently cerebral perfusion pressure (CPP) with detrimental consequences on cerebral blood flow (CBF), mainly in cases of altered cerebral autoregulation [16]. On the other hand, respiratory system compliance influences the effect of PEEP on ICP and brain circulation [17–19]: in patients with low compliance, a PEEP increase (up to 10–12 cmH_2_O) is not associated with ICP increase; in contrast, in patients with normal compliance, PEEP induces lung overdistension, reduction in cerebral venous return and ICP increases. We found that most of our respondents utilize lower PEEP levels in case of intracranial hypertension, thus suggesting that there is still concern regarding the cerebrovascular effects of PEEP on ICP. However, surprisingly, our results show that physicians are keen to use quite high PEEPs (up to 15 in patients with PaO_2_/FiO_2_ > 300) in patients without intracranial hypertension, which is in contrast with growing evidence challenging the utility of “open lung approach” [20]. Probably, the availability of adequate neuro and cardiorespiratory monitoring tools could be useful in setting the right level of PEEP, but more prospective randomized studies are needed to identify the correct level of PEEP in head-injured patients.

Data from our survey show that hyperventilation is not often utilized in TBI patients. This is probably related to the potential ischemic side effects of hypocapnia on the injured brain [21]. Currently, hyperventilation is suggested only in case of emergency with life-threatening risk of cerebral herniation [22]. However, recent findings suggested that mild, short-term hyperventilation is able to reduce ICP, without a clinically significant reduction of cerebral oxygenation and metabolism [23]. In fact, most responders aimed to a target of 36–40 mmHg of CO_2_, and only a minority accept mild hypercapnia, even in the absence of intracranial hypertension.

Regarding PaO_2_ level, the majority of respondents chose a target of 81–100 mmHg, greater than those used in ARDS patients (55–80 mmHg) and just a minority of respondents accept mild hypoxemia, especially in non-specialized NICU centres [12]. This choice could be linked to the concern of hypoxia and its detrimental effects on brain-injured patients, but, on the other hand, to the recent evidence regarding the risk related to hyperoxia on critically ill patients [24–27].

### Rescue strategies for refractory respiratory failure

In case of refractory respiratory failure, several rescue manoeuvres are generally employed as adjunct to invasive mechanical ventilation to ameliorate gas exchange. NMBAs have been utilized in ARDS patients to improve gas exchange with inconclusive effects on mortality [28, 29]. Neuromuscular blockade is the most frequently used rescue strategy by our respondents; this can be related to their possible beneficial effect also on intracranial hypertension [30, 31]. However, the utilization of NMBAs should take into account their side effects (such as muscle weakness) and should be reserved to the most severe cases [30].

Recruitment manoeuvres can reduce atelectasis and increase lung expiratory lung volumes with potential benefit on gas exchange and lung injury [32]. However, their use has been recently questioned by a recent study showing an increase in mortality in ARDS patients undergoing lung recruitment manoeuvres and titrated PEEP (to the best respiratory system compliance) vs. low PEEP [33]. Recruitment manoeuvres can have potential side effects on the injured brain causing increased intrathoracic pressure similar to the application of high level of PEEP [34]. Despite these effects, they represent the second most utilized rescue strategy in our survey. Data are lacking about the optimal recruitment strategy in TBI patients with ARDS but in this context the use of neuromonitoring (i.e. ICP) seems to be mandatory to optimize systemic oxygenation without causing side effects.

Prone position is frequently utilized in ARDS patients because of its ability to improve ventilation/perfusion ratio, to increase end-expiratory lung volume and to decrease VILI by ameliorating the distribution of the TV [32]. This manoeuvre is able to ameliorate mortality in ARDS only if applied for more than 12 h/day and in patients with a PaO_2_/FiO_2_ < 150 [32]. Small studies have been published so far involving patients with neurological damage; in this situation, despite an improvement in systemic/brain oxygenation, an increase in ICP has been often observed [35–39]. As consequence, patients with brain injury have been generally excluded from the only available trial on prone position on ARDS patients [13].

Recently, extracorporeal membrane oxygenation (ECMO) has been utilized as a rescue strategy in ARDS patients [39, 40]. Historically, ECMO was not utilized in TBI patients because of the risk of cerebral bleeding related to the use of anticoagulation. Recently, some case reports and small case series have suggested a potential beneficial use of ECMO in TBI patients [41]. Surprisingly, nearly half of our respondents would use ECMO in TBI patients as rescue respiratory strategy. This could be related to the possibility to adopt heparin-free veno-venous (VV) ECMO with consequent less risk of bleeding complications [42, 43]. However, the role of ECMO in TBI patients with refractory respiratory therapy requires further investigations.

Finally, only a small amount of our respondents suggested the use of extracorporeal CO_2_ removal (i.e. DECAP®) in TBI patients; although a small dose of heparin is necessary for this method with low risk of bleeding, the efficacy of CO_2_ extracorporeal CO_2_ removal is limited and rarely used in the clinical practice [44].

Ventilatory strategies in trauma patients could take into account the phase of treatment and the underlying injuries [45].

### Limitations

As with all methods of data collection, survey research also comes with a few drawbacks as inflexibility and validity. This survey presents also other limitations. First, the response rate cannot be calculated considering the design of this survey; in fact, ESICM members are invited to involve more participants locally, thus making impossible to obtain the total number of people who received the survey. Second, respondents from specialized NICUs were included together with those working in general ICUs. This might be considered a methodological limitation but, as TBI patients are treated worldwide not only in specialized NICUs, we believe that our approach produced more generalizable findings. Third, this survey was developed by two investigators without a prior systematic review of the literature or pilot testing in a smaller sample of participants; this might further limit the quality of the questionnaire and data. Fourth, this survey refers only to physicians’ clinical practice in respiratory management of TBI without including patients’ data.

## Conclusions

In conclusion, this survey shows that important differences in the clinical practice in the ventilatory management of TBI patients with and without respiratory failure still exist. Lung-protective ventilation strategies seem to be more frequently applied in the clinical practice in brain-injured patients, although most respondents still seem to have concerns regarding the use of high PEEP in case of intracranial hypertension. Future studies are warranted to clarify the huge practice variability among centres.

## Supplementary information

Additional file 1. Appendix 1, Table S1, Table S2, − Table S3. (DOCX 40 kb)

## Abbreviations

TBI: Traumatic brain injury
ARDS: Acute respiratory distress syndrome
PaO_2_: Arterial partial pressure of oxygen
FiO_2_: Inspired fraction of oxygen
ESICM: European Society of Intensive Care Medicine
PBW: Predicted body weight
PEEP: Positive end-expiratory pressure
ICU: Intensive care unit
TV: Tidal volume
CO_2_: Carbon dioxide
NICU: Neuro-ICU
PaCO_2_: Arterial partial pressure of CO_2_
NMBAs: Neuromuscular blocking agent
ICP: Intracranial pressure
CPP: Cerebral perfusion pressure
CBF: Cerebral blood flow
ECMO: Extracorporeal membrane oxygenation
VV: Veno-venous
NIRS: Near-infrared spectroscopy
