# Supplementary material for: VENTILatOry strategies in patients with severe traumatic brain injury: the VENTILO Survey of the European Society of Intensive Care Medicine (ESICM)
Source: Crit Care. 2020 Apr 17;24:158. doi: 10.1186/s13054-020-02875-w (PMC7165367; doi:10.1186/s13054-020-02875-w)
Supplement: Supplementary file 1 — Appendix 1, Table S1, Table S2, − Table S3. (DOCX 40 kb) [file 13054_2020_2875_MOESM1_ESM.docx]

**ADDITIONAL FILE 1**

**Appendix - 1.** Survey questionnaire.

1) Gender:

- female

- male

2) Age (years):

- < 35

- 35-45

- 45-60

- > 60

3) In which country do you work ?

4) Affiliation:

- university/academic

- non academic hospital

5) Professional category:

- doctor

- resident

- nurse

- allied health care professional

- other (please specify)

6)Post-specialization experience in critical care (years):

- 1-5

- 6-10

- > 10

7) Specialty:

- anaesthesiologist

- intensive care

- neurocritical care

- neurosurgeon

- neurologist

- internal medicine

- pneumology

- other (please specify)

8) Numbers of beds in your ICU with neuro patients (general or specialized):

- < 5

- 6 - 10

- 11 - 15

- > 15

9) Type of intensive care unit (ICU):

- general ICU

- specialized neuroICU

- mixed general and neuroICU

- other (please specify)

10) Type of bedside neuromonitoring available in your unit (allowed more than one answers):

- intracranial pressure

- PbtO2

- NIRS

- transcranial doppler

- cerebral microdialysis

- intermittent EEG

- continuous EEG

- automated pupillometry

- SjVO2

11) Do you currently use in your clinical practice a standard protocol for mechanical ventilation in TBI patients ?

- yes

- no

11-a) If YES, is this based for specific setting for (allowed more than one answers):

- tidal volume

- PEEP

- FiO2

- RR

- driving pressure

- plateau pressure

- transpulmonary pressure

12) Do you currently use automated ventilation modes ?

- yes

- no

13) Do you currently use a specific weaning protocol in patients with TBI ?

- yes

- no

13-a) If YES, which are the specific setting on which it is based ?

- pH

- GCS

- PaO2/FiO2

- PaO2

- PaCO2

- swallowing

14) Do you target mechanical ventilation to driving pressure < 15 cmH2O ?

- yes

- no

15) Do you target mechanical ventilation to plateau pressure < 30 cmH2O ?

- yes

- no

**Clinical Scenario 1.** You have to set up the ventilator for a 45-year old man admitted to your ICU with a severe traumatic brain injury (TBI) [i.e. Glasgow coma scale (GCS) < 9 and different abnormalities on cerebral computed tomography (CT)]. The patients is deeply sedated and there is no triggering for the ventilation.

Ventilatory targets in TBI patients with PaO2/FIO2 > 300.

16) Which is the tidal volume target that you use in your clinical practice ?

- 4-6 ml of predicted body weight (PBW)

- 6-8 ml of PBW

- 8-10 ml of PBW

- other (please specify)

17) Which is the highest value of PEEP that you would set if the patient has not intracranial hypertension ?

- 5 cmH2O

- 8 cmH2O

- 10 cm H2O

- 15 cmH2O

- other (please specify)

18) Which is the highest value of PEEP that you would set if the patient has intracranial hypertension ?

- 5 cmH2O

- 8 cmH2O

- 10 cm H2O

- 15 cmH2O

- other (please specify)

19) Which is the PaCO2 target that you use in the clinical practice without intracranial hypertension ?

- 30-35 mmHg

- 36-40 mmHg

- 41-45 mmHg

- 46-55 mmHg

- any PaCO2 if pHa is in range

- other (please specify)

20) Which is the PaCO2 target that you use in the clinical practice in a patient with TBI and intracranial hypertension ?

- 30-35 mmHg

- 36-40 mmHg

- 41-45 mmHg

- 46-55 mmHg

- any PaCO2 if pHa is in range

- other (please specify)

21) Which is the PaO2 target that you sue in your clinical practice ?

- 55-80 mmHg

- 81-100 mmHg

- 101-120 mmHg

- > 120 mmHg

- adjusted on neuromonitoring

22) Which is the SpO2 target that you use in your clinical practice ?

- 88-91%

- 92-84%

- > 95%

**Clinical Scenario 2.** You have to set up the ventilator for a 45-year old man admitted to your ICU with a severe traumatic brain injury (TBI) [i.e. Glasgow coma scale (GCS) < 9 and different abnormalities on cerebral computed tomography (CT)]. The patients is deeply sedated and there is no triggering for the ventilation.

Ventilatory targets in TBI patients with PaO2/FIO2 150-300.

23) Which is the tidal volume target that you use in your clinical practice ?

- 4-6 ml of predicted body weight (PBW)

- 6-8 ml of PBW

- 8-10 ml of PBW

- other (please specify)

24) Which is the highest value of PEEP that you would set if the patient has not intracranial hypertension ?

- 5 cmH2O

- 8 cmH2O

- 10 cm H2O

- 15 cmH2O

- other (please specify)

25) Which is the highest value of PEEP that you would set if the patient has intracranial hypertension ?

- 5 cmH2O

- 8 cmH2O

- 10 cm H2O

- 15 cmH2O

- other (please specify)

26) Which is the PaCO2 target that you use in the clinical practice without intracranial hypertension ?

- 30-35 mmHg

- 36-40 mmHg

- 41-45 mmHg

- 46-55 mmHg

- any PaCO2 if pHa is in range

- other (please specify)

27) Which is the PaCO2 target that you use in the clinical practice in a patient with TBI and intracranial hypertension ?

- 30-35 mmHg

- 36-40 mmHg

- 41-45 mmHg

- 46-55 mmHg

- any PaCO2 if pHa is in range

- other (please specify)

28) Which is the PaO2 target that you sue in your clinical practice ?

- 55-80 mmHg

- 81-100 mmHg

- 101-120 mmHg

- > 120 mmHg

- adjusted on neuromonitoring

29) Which is the SpO2 target that you use in your clinical practice ?

- 88-91%

- 92-84%

- > 95%

**Clinical Scenario 3.** You have to set up the ventilator for a 45-year old man admitted to your ICU with a severe traumatic brain injury (TBI) [i.e. Glasgow coma scale (GCS) < 9 and different abnormalities on cerebral computed tomography (CT)]. The patients is deeply sedated and there is no triggering for the ventilation.

Ventilatory targets in TBI patients with PaO2/FIO2 < 150.

30) Which is the tidal volume target that you use in your clinical practice ?

- 4-6 ml of predicted body weight (PBW)

- 6-8 ml of PBW

- 8-10 ml of PBW

- other (please specify)

31) Which is the highest value of PEEP that you would set if the patient has not intracranial hypertension ?

- 5 cmH2O

- 8 cmH2O

- 10 cm H2O

- 15 cmH2O

- other (please specify)

32) Which is the highest value of PEEP that you would set if the patient has intracranial hypertension ?

- 5 cmH2O

- 8 cmH2O

- 10 cm H2O

- 15 cmH2O

- other (please specify)

33) Which is the PaCO2 target that you use in the clinical practice without intracranial hypertension ?

- 30-35 mmHg

- 36-40 mmHg

- 41-45 mmHg

- 46-55 mmHg

- any PaCO2 if pHa is in range

- other (please specify)

34) Which is the PaCO2 target that you use in the clinical practice in a patient with TBI and intracranial hypertension ?

- 30-35 mmHg

- 36-40 mmHg

- 41-45 mmHg

- 46-55 mmHg

- any PaCO2 if pHa is in range

- other (please specify)

35) Which is the PaO2 target that you sue in your clinical practice ?

- 55-80 mmHg

- 81-100 mmHg

- 101-120 mmHg

- > 120 mmHg

- adjusted on neuromonitoring

36) Which is the SpO2 target that you use in your clinical practice ?

- 88-91%

- 92-84%

- > 95%

37) Which of the following ventilatory rescue strategies would you consider in a patient with TBI and refractory respiratory failure ?

- inhaled nitric oxide (NO)

- prostacycline

- bronchoscopy

- neuromuscular blocking agents (NMBAs)

- recruitment maneuvers

- prone position

- extracorporeal CO2 removal (DECAP®)

- veno-venous extracorporeal membrane oxygenation (V-V ECMO)

38) In which order would you consider the previous strategies ? (score 1 to 8)

**Table S1.** The number of responders related to completed items.

| Item’s Number | Number of Responders |
| --- | --- |
| 1-11 | 687 |
| 11a | 277 |
| 12 | 668 |
| 13 | 668 |
| 13a | 198 |
| 14-15 | 646 |
| 16-22 (cs 1) | 601 |
| 23-29 (cs 2) | 536 |
| 30 (cs 3) | 480 |
| 31-32 (cs 3) | 481 |
| 33 (cs 3) | 480 |
| 34 (cs 3) | 481 |
| 35 (cs 3) | 480 |
| 36 (cs 3) | 481 |
| 37 | 464 |
| 38 | 636 |

Abbreviations: cs = clinical scenario.

**Table S-2:** Comparisons between European (EU) vs. non-European (Non-EU) countries and between non-specialized (other ICUs) vs specialized neuro-ICUs (NICU).

|  |  | **Non-Eu** | **Eu** | **P** |  | **Other ICUs** | **NICU** | **P** |
| --- | --- | --- | --- | --- | --- | --- | --- | --- |
| **Protocol for MV in TBI** | |  |  |  |  |  |  |  |
|  | No | 130(49) | 280(67) | < 0.001 |  | 335(60) | 75(58) | 0.61 |
|  | Yes | 137(51) | 144(33) |  |  | 222(40) | 55(42) |  |
|  |  |  | |  |  |  | |  |
|  |  |  |  |  |  |  |  |  |
| **Weaning protocol in TBI** | |  |  |  |  |  |  |  |
|  | No | 147(57) | 323(79) | < 0.001 |  | 388(71) | 82(66) | 0.25 |
|  | Yes | 110(43) | 88(21) |  |  | 156(29) | 42(34) |  |
|  |  |  | |  |  |  | |  |
|  |  |  |  |  |  |  |  |  |
| **DP < 15 cmH_2_O** | |  |  |  |  |  |  |  |
|  | No | 66(27) | 144(36) | 0.02 |  | 159(30) | 51(42) | 0.001 |
|  | Yes | 180(73) | 256(64) |  |  | 366(70) | 70(58) |  |
|  |  |  | |  |  |  | |  |
|  |  |  |  |  |  |  |  |  |
| **Pplat < 30 cmH_2_O** | |  |  |  |  |  |  |  |
|  | No | 7(3) | 23(6) | 0.09 |  | 13(11) | 17(3) | < 0.001 |
|  | Yes | 239(97) | 377(94) |  |  | 108(89) | 508(97) |  |
|  |  |  | |  |  |  | |  |
|  |  |  |  |  |  |  |  |  |
| **TV (ml/kg PBW)**  **CS-1** | | |  |  |  |  |  |  |
|  | 4-6 | 59(26) | 77(21) | 0.15 |  | 118(24) | 18(16) | 0.02 |
|  | 6-8 | 163(71) | 270(73) |  |  | 353(72) | 80(73) |  |
|  | 8-10 | 5(2) | 16(4) |  |  | 13 (3) | 8(7) |  |
|  | other | 2(1) | 9(2) |  |  | 7(1) | 4(4) |  |
|  |  |  |  |  |  |  |  |  |
| **Highest PEEP no-IH (cmH_2_O)**  **CS-1** | | |  |  |  |  |  |  |
|  | 5 | 31(13) | 38(10) | 0.18 |  | 52(10) | 17(15) | 0.72 |
|  | 8 | 43(19) | 51(14) |  |  | 81(17) | 13(12) |  |
|  | 10 | 59(26) | 99(27) |  |  | 131(27) | 27(24) |  |
|  | 15 | 66(29) | 116(31) |  |  | 146(30) | 36(33) |  |
|  | other | 30(13) | 68(18) |  |  | 81(16) | 17(16) |  |
| **Highest PEEP with IH (cmH_2_O)**  **CS-1** | | | | | | | | |
|  | 5 | 56(24) | 107(29) | 0.08 |  | 133(27) | 30(27) | 0.89 |
|  | 8 | 61(27) | 74(20) |  |  | 114(23) | 21(19) |  |
|  | 10 | 66(29) | 89(24) |  |  | 126(26) | 29(27) |  |
|  | 15 | 16(7) | 36(10) |  |  | 42(9) | 10(9) |  |
|  | other | 30(13) | 66(17) |  |  | 76(15) | 20(18) |  |
|  |  |  |  |  |  |  |  |  |
|  |  | **Non-Eu** | **Eu** | **P** |  | **Other ICUs** | **NICU** | **P** |
| **PaCO_2_ no-IH (mmHg)**  **CS-1** | | | | | | | | |
|  |  |  |  |  |  |  |  |  |
|  | 30-35 | 26(11) | 28(7) | 0.13 |  | 45(10) | 9(9) | 0.42 |
|  | 36-40 | 127(56) | 181(49) |  |  | 260(55) | 48(47) |  |
|  | 41-45 | 32(14) | 73(20) |  |  | 81(17) | 24(23) |  |
|  | 46-55 | 5(2) | 11(3) |  |  | 13(3) | 3(3) |  |
|  | any CO2* | 29(13) | 65(17) |  |  | 74(15) | 19(18) |  |
|  | other | 10(4) | 15(4) |  |  | 0(0) | 0(0) |  |
|  |  |  |  |  |  |  |  |  |
| **PaCO_2_ with IH (mmHg)**  **CS-1** | | | | | | | | |
|  |  |  |  |  |  |  |  |  |
|  | 30-35 | 121(53) | 128(34) | < 0.01 |  | 209(43) | 40(36) | 0.08 |
|  | 36-40 | 77(34) | 183(49) |  |  | 214(44) | 46(42) |  |
|  | 41-45 | 14(6) | 28(8) |  |  | 33(7) | 9(8) |  |
|  | 46-55 | 1(1) | 7(2) |  |  | 7(1) | 1(1) |  |
|  | any CO2* | 5(2) | 2(1) |  |  | 6(1) | 1(1) |  |
|  | other | 11(4) | 24(6) |  |  | 22(4) | 13(12) |  |
|  |  |  |  |  |  |  |  |  |
| **PaO_2_ target (mmHg)**  **CS-1** | | | |  |  |  |  |  |
|  | 55-88 | 58(25) | 43(12) | < 0.001 |  | 95(19) | 6(6) | 0.005 |
|  | 81-100 | 132(58) | 213(57) |  |  | 279(57) | 66(60) |  |
|  | 101-120 | 29(13) | 64(17) |  |  | 71(15) | 22(20) |  |
|  | > 120 | 5(2) | 13(4) |  |  | 13(3) | 5(5) |  |
|  | nmt** | 5(2) | 39(10) |  |  | 33(6) | 11(19) |  |
|  |  |  | |  |  |  | |  |
| **SpO_2_ target (%)**  **CS-1** | | | | | | | | |
|  |  |  |  |  |  |  |  |  |
|  | 88-91 | 20(9) | 13(4) | < 0.001 |  | 32(7) | 1(1) | < 0.001 |
|  | 92-94 | 118(52) | 139(37) |  |  | 231(47) | 26(24) |  |
|  | > 95 | 91(39) | 220(59) |  |  | 228(46) | 83(75) |  |
|  |  |  |  |  |  |  |  |  |
| **TV (ml/kg PBW)**  **CS-2** | |  |  |  |  |  |  |  |
|  | 4-6 | 87(43) | 105(31) | 0.02 |  | 162(37) | 30(31) | 0.62 |
|  | 6-8 | 112(56) | 219(65) |  |  | 266(60) | 65(66) |  |
|  | 8-10 | 0(0) | 3(1) |  |  | 2(1) | 1(1) |  |
|  | other | 2(1) | 8(3) |  |  | 8(2) | 2(2) |  |
|  |  |  |  |  |  |  |  |  |
| **Highest PEEP no-IH (cmH_2_O)**  **CS-2** | | | | | | | | |
|  | 5 | 13(7) | 10(3) | 0.2 |  | 14(3) | 9(9) | 0.04 |
|  | 8 | 29(14) | 42(13) |  |  | 61(14) | 10(11) |  |
|  | 10 | 42(21) | 94(28) |  |  | 117(27) | 19(19) |  |
|  | 15 | 83(41) | 135(40) |  |  | 173(40) | 45(46) |  |
|  | other | 34(17) | 54(16) |  |  | 73(16) | 15(15) |  |
|  |  |  |  |  |  |  |  |  |
|  |  | **Non-Eu** | **Eu** | **P** |  | **Other ICUs** | **NICU** | **P** |
| **Highest PEEP with IH (cmH_2_O)**  **CS-2** | | | | | | | | |
|  | 5 | 34(17) | 69(21) | 0.08 |  | 86(20) | 17(17) | 0.6 |
|  | 8 | 51(25) | 80(24) |  |  | 112(26) | 19(19) |  |
|  | 10 | 77(38) | 94(28) |  |  | 136(31) | 35(37) |  |
|  | 15 | 16(9) | 38(11) |  |  | 44(10) | 10(10) |  |
|  | other | 23(11) | 54(16) |  |  | 60(13) | 17(17) |  |
|  |  |  |  |  |  |  |  |  |
| **PaCO_2_ no-IH (mmHg)**  **CS-2** | | | | | | | | |
|  | 30-35 | 18(9) | 16(5) | 0.005 |  | 30(7) | 4(4) | 0.3 |
|  | 36-40 | 108(54) | 142(42) |  |  | 206(47) | 44(46) |  |
|  | 41-45 | 28(14) | 82(25) |  |  | 93(21) | 17(17) |  |
|  | 46-55 | 12(6) | 23(7) |  |  | 27(6) | 8(8) |  |
|  | any CO2* | 31(15) | 56(17) |  |  | 69(16) | 18(18) |  |
|  | other | 4(2) | 16(4) |  |  | 13(3) | 7(7) |  |
|  |  |  |  |  |  |  |  |  |
| **PaCO_2_ with IH (mmHg)**  **CS-2** | | | | | | | | |
|  | 30-35 | 85(42) | 91(27) | 0.007 |  | 147(34) | 29(30) | 0.1 |
|  | 36-40 | 90(45) | 172(51) |  |  | 217(50) | 45(46) |  |
|  | 41-45 | 14(7) | 40(12) |  |  | 44(9) | 10(10) |  |
|  | 46-55 | 1(1) | 3(1) |  |  | 4(1) | 0(0) |  |
|  | any CO2* | 4(2) | 6(2) |  |  | 7(2) | 3(3) |  |
|  | other | 7(3) | 23(7) |  |  | 19(4) | 11(11) |  |
|  |  |  |  |  |  |  |  |  |
| **PaO_2_ target (mmHg)**  **CS-2** | | | | | | | | |
|  | 55-80 | 59(29) | 56(17) | 0.001 |  | 105(24) | 10(10) | 0.001 |
|  | 81-100 | 103(51) | 180(54) |  |  | 231(53) | 52(54) |  |
|  | 101-120 | 27(13) | 57(17) |  |  | 63(14) | 21(21) |  |
|  | > 120 | 7(4) | 12(4) |  |  | 14(3) | 5(5) |  |
|  | nmt** | 5(3) | 30(8) |  |  | 25(6) | 10(10) |  |
|  |  |  |  |  |  |  |  |  |
| **SpO_2_ target (%)**  **CS-2** | | | | | | | | |
|  | 88-91 | 33(16) | 17(5) | < 0.001 |  | 48(11) | 2(2) | < 0.001 |
|  | 92-94 | 106(53) | 152(45) |  |  | 223(51) | 35(36) |  |
|  | > 95 | 62(31) | 166(50) |  |  | 167(38) | 61(62) |  |
|  |  |  |  |  |  |  |  |  |
| **TV (ml/kg PBW)**  **CS-3** | | | | | | | | |
|  | 4-6 | 112(62) | 140(47) | 0.003 |  | 215(55) | 37(40) | 0.004 |
|  | 6-8 | 62(34) | 141(47) |  |  | 160(41) | 43(48) |  |
|  | 8-10 | 4(2) | 5(2) |  |  | 4(1) | 5(6) |  |
|  | other | 2(2) | 14(4) |  |  | 11(3) | 5(6) |  |
|  |  |  |  |  |  |  |  |  |
|  |  | **Non-Eu** | **Eu** | **P** |  | **Other ICUs** | **NICU** | **P** |
| **Highest PEEP no-IH (cmH_2_O)**  **CS-3** | | | | | | | | |
|  | 5 | 9(5) | 5(2) | 0.2 |  | 9(2) | 5(6) | 0.2 |
|  | 8 | 17(9) | 20(7) |  |  | 34(9) | 3(3) |  |
|  | 10 | 33(18) | 62(21) |  |  | 75(19) | 20(22) |  |
|  | 15 | 85(47) | 154(50) |  |  | 195(50) | 44(49) |  |
|  | other | 36(21) | 60(20) |  |  | 78(20) | 18(20) |  |
|  |  |  |  |  |  |  |  |  |
| **Highest PEEP with IH (cmH_2_O)**  **CS-3** | | | | | | | | |
|  | 5 | 20(11) | 35(12) | 0.1 |  | 46(12) | 9(10) | 0.4 |
|  | 8 | 31(17) | 63(21) |  |  | 82(21) | 12(13) |  |
|  | 10 | 73(41) | 85(28) |  |  | 126(32) | 32(36) |  |
|  | 15 | 29(16) | 62(21) |  |  | 74(19) | 17(19) |  |
|  | other | 27(15) | 56(18) |  |  | 63(16) | 20(22) |  |
|  |  |  |  |  |  |  |  |  |
| **PaCO_2_ no-IH (mmHg)**  **CS-3** | | | | | | | | |
|  | 30-35 | 19(11) | 10(3) | 0.02 |  | 27(6) | 2(2) | 0.4 |
|  | 36-40 | 69(38) | 106(35) |  |  | 147(38) | 28(31) |  |
|  | 41-45 | 40(22) | 78(26) |  |  | 92(24) | 26(29) |  |
|  | 46-55 | 18(10) | 30(10) |  |  | 38(10) | 10(11) |  |
|  | any CO2* | 30(17) | 61(20) |  |  | 72(19) | 19(21) |  |
|  | other | 4(2) | 15(6) |  |  | 4(3) | 5(6) |  |
|  |  |  |  |  |  |  |  |  |
| **PaCO_2_ with IH (mmHg)**  **CS-3** | | | | | | | | |
|  | 30-35 | 62(35) | 67(22) | 0.02 |  | 113(28) | 16(18) | 0.03 |
|  | 36-40 | 77(43) | 147(49) |  |  | 184(47) | 40(44) |  |
|  | 41-45 | 22(12) | 53(18) |  |  | 57(15) | 18(21) |  |
|  | 46-55 | 4(2) | 5(2) |  |  | 8(2) | 1(1) |  |
|  | any CO2* | 8(4) | 7(2) |  |  | 11(3) | 4(4) |  |
|  | other | 7(4) | 22(7) |  |  | 18(5) | 11(12) |  |
|  |  |  |  |  |  |  |  |  |
| **PaO_2_ target (mmHg)**  **CS-3** | | | | | | | | |
|  | 55-80 | 73 (41) | 78(26) | 0.003 |  | 128(33) | 23(26) | 0.02 |
|  | 81-100 | 72(40) | 146(49) |  |  | 184(47) | 34(38) |  |
|  | 101-120 | 24(13) | 37(12) |  |  | 42(11) | 19(21) |  |
|  | > 120 | 5(3) | 9(3) |  |  | 11(3) | 3(3) |  |
|  | nmt** | 6(3) | 30(10) |  |  | 25(6) | 11(12) |  |
|  |  |  |  |  |  |  |  |  |
| **SpO_2_ target (%)**  **CS-3** | | | | | | | | |
|  | 88-91 | 58(32) | 48(16) | < 0.001 |  | 97(25) | 9(10) | 0.002 |
|  | 92-94 | 84(47) | 143(48) |  |  | 184(47) | 43(48) |  |
|  | > 95 | 38(21) | 110(36) |  |  | 110(28) | 38(42) |  |
|  |  | **Non-Eu** | **Eu** | **P** |  | **Other ICUs** | **NICU** | **P** |
| **Rescue strategies for RRF** | | | | | | | | |
|  | NO | 24(15) | 90(31) | < 0.001 |  | 98(26) | 17(19) | 0.2 |
|  | prostacycline | 13(8) | 25(8) | 0.72 |  | 27(7) | 11(13) | 0.1 |
|  | bronchoscopy | 57(33) | 182(62) | <0.001 |  | 183(49) | 56(64) | 0.01 |
|  | NMBAs | 137(80) | 269(92) | <0.001 |  | 329(88) | 77(88) | 1 |
|  | RM | 107(63) | 212(72) | 0.003 |  | 251(67) | 68(77) | 0.06 |
|  | PP | 103(60) | 189(65) | 0.36 |  | 236(63) | 56(64) | 0.9 |
|  | DECAP® | 5(3) | 34(12) | 0.001 |  | 32(9) | 7(8) | 0.9 |
|  | V-V ECMO | 89(52) | 131(45) | 0.13 |  | 179(48) | 41(47) | 0.9 |
|  |  |  | |  |  |  | |  |

* if arterial pH is in range

** adjusted to

Legend – number (percentage); EU = european countries; Non-Eu = non-european countries; ICU = intensive care unit; NICU = neuroICU; MV = mechanical ventilation, TBI = traumatic brain injury; DP = driving pressure; Pplat = plateau pressure; Tv = tidal volume; CS = case scenario; PEEP = positive end-expiratory pressure; IH = intracranial hypertension; PaCO2 = partial pressure of arterial carbon dioxide; PaO2 = partial pressure of arterial oxygen; SpO2 = arterial blood oxygen saturation; nmt = neuromonitoring; NO = nitric oxide; NMBA = neuromuscular blocking agent; RM = recruitment manoeuvre; PP = prone position; V-V ECMO = veno-venous extracorporeal membrane oxygenation; RRF = refractory respiratory failure.

**Table S-3:** Comparisons of available bedside neuromonitoring between non-specialized (other ICUs) vs specialized neuro-ICUs (NICU)

| Available bedside neuromonitoring | |  | **Other ICUs** | **NICU** | **P** |
| --- | --- | --- | --- | --- | --- |
|  |  |  |  |  |  |
|  | ICP |  | 430(77) | 116(89) | 0.002 |
|  | PbtO_2_ |  | 86(15) | 63(49) | <0.001 |
|  | NIRS |  | 176(32) | 39(30) | 0.724 |
|  | TCD |  | 333(60) | 109(84) | <0.001 |
|  | MD |  | 23(4) | 27(21) | <0.001 |
|  | EEG |  | 414(74) | 108(83) | 0.035 |
|  | Cont. EEG |  | 177(32) | 85(65) | <0.001 |
|  | Aut. Pupill. |  | 40(7) | 45(35) | <0.001 |
|  | SjVO_2_ |  | 158(28) | 51(39) | 0.015 |
|  |  |  |  |  |  |

Legend – number (percentage); ICU = intensive care unit, NICU = neuroICU, ICP = intracranial pressure, PbtO_2_ = brain tissue oxygenation, NIRS = near-infrared spectroscopy, TCD = transcranial doppler, MD = microdialysis, EEG = electroencephalography, Cont. EEG = continuous EEG, Aut. Pupill. = automated pupillometry, SjVO_2_ = jugular venous oxygen saturation.
